# The plant hopper *Issus coleoptratus* can detoxify phloem sap saponins including the degradation of the terpene core

**DOI:** 10.1242/bio.016311

**Published:** 2016-02-10

**Authors:** Markus Himmelsbach, Agnes Weth, Christine Böhme, Martin Schwarz, Peter Bräunig, Werner Baumgartner

**Affiliations:** 1 Institute of Analytical Chemistry, Johannes Kepler University Linz, Altenbergerstr. 69, Linz 4040, Austria; 2 Institute of Biomedical Mechatronics, Johannes Kepler University Linz, Altenbergerstr. 69, Linz 4040, Austria; 3 Institute of Biology 2 (Zoology), RWTH-Aachen University, Worringerweg 3, Aachen 52056, Germany; 4 Biology Center Linz, Johann-Wilhelm-Klein-Straße 73, Linz 4040, Austria

**Keywords:** Fulgoromorpha, Ivy, Terpene, Genine, *Hedera*, Mass spectroscopy

## Abstract

*Issus coleoptratus* is a small plant hopper which mainly feeds on the phloem sap from ivy. Although all parts of ivy are poisonous as the plant contains saponins, especially hederasaponins, *I.* *coleoptratus* can cope with the poison. In contrast to other animals like the stick insect *Carausius morosus* which accumulates saponins in its body, *I. coleoptratus* can degrade and disintegrate not only the saponins but even the genines, i.e. the triterpene core of the substances. This is perhaps made possible by a specialised midgut and/or the salivary glands. When the glands and the gut are dissected and added to saponins in solution, the saponins, including the genines, are degraded *ex vivo*.

## Abstract (precis)

**Summary:** In contrast to other insects the plant hopper *Issus coleoptratus* does not accumulate toxic saponins from ivy. *I. coleoptratus* can degrade the saponines by its salivary glands and its gut as shown by HPLC-MS.

## INTRODUCTION

The planthopper *Issus coleoptratus* (Fabricius, 1781) is a common insect species in the western Palaearctic (Strümpel, 2014). It reaches a body length of about 5.5–7.0 mm in the adult stage and has a rather compact, stocky build. There is only one generation per year and the total life span may be just over one year (Strümpel, 2014). It feeds on the phloem sap of different plants. We found *I. coleoptratus* almost exclusively on ivy (*Hedera*), but according to the literature (Remane and Wachmann, 1993; Holzinger et al., 2003) the insects also feed on juniper (*Juniperus*), yew (*Taxus*), privet (*Ligustrum*) and English Holly (*Ilex*), especially during the winter. However, the latter two were not accepted as food plants by these insects in captivity. It is striking that all these plants contain poisonous terpenes which may act as defensive substances. Ivy, for instance, contains significant amounts of saponins, especially hederines (α-hederin, β-hederin, δ-hederin, hederasaponin C) as defensive substances. Saponins are a class of chemical compounds found in particular abundance in various plant species (Seki et al., 2015) and are discussed as potential agents for pest control. They are amphipathic glycosides grouped phenomenologically by the soap-like foaming they produce when shaken in aqueous solutions, and structurally by having one or more hydrophilic glycoside moieties combined with a lipophilic triterpene derivative.

In the present study we investigate the morphology of the *I.* *coleoptratus* digestive tract and how these insects can cope with saponins.

## RESULTS AND DISCUSSION

**Fig. 1.**
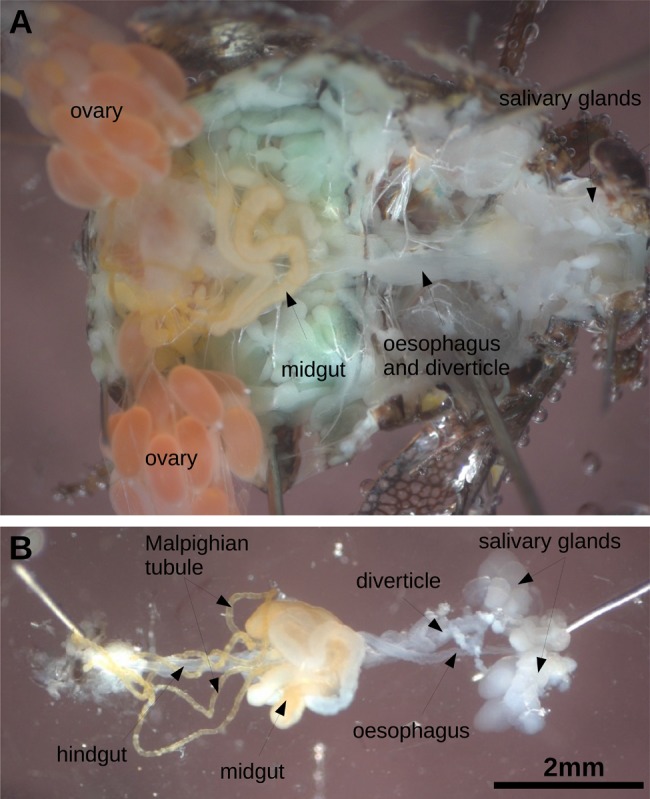
***The gut of*** ***I.*** ***coleoptratus*.** (A) A specimen of total length of 6 mm opened dorsally. The ovaries are deflected laterally to expose the gut. (B) The gut and salivary glands dissected from the specimen shown in A.

The anatomy of the inner organs of *I.* *coleoptratus* is similar to other plant hoppers (Fick, 1983; Strümpel, 2014) but one feature appears special (Fig. 1): the midgut is strongly convoluted and very long. After unwinding the coils of the midgut shown in Fig. 1B the total length of the gut was 27 mm while the corresponding animal (Fig. 1A) had a length of only 6 mm. To the best of our knowledge such a long midgut has not yet been described for any insect, although a somewhat convoluted part of the midgut forming a so-called filter chamber was previously described for other plant hoppers (Strümpel, 2014). For this reason we asked ourselves why an animal feeding on ‘sugar water’ would need such a long digestive system, because in other plant-feeding insects such as locusts or stick insects the gut is only 1-1.5 times the length of the body. The same holds true for fruit flies (personal observations).

We found *I.* *coleoptratus* almost exclusively on ivy and could raise them from hatching through all nymphal stages to adult with ivy being the only food source, which also appears to be their favourite plant in our region.

**Fig. 2.**
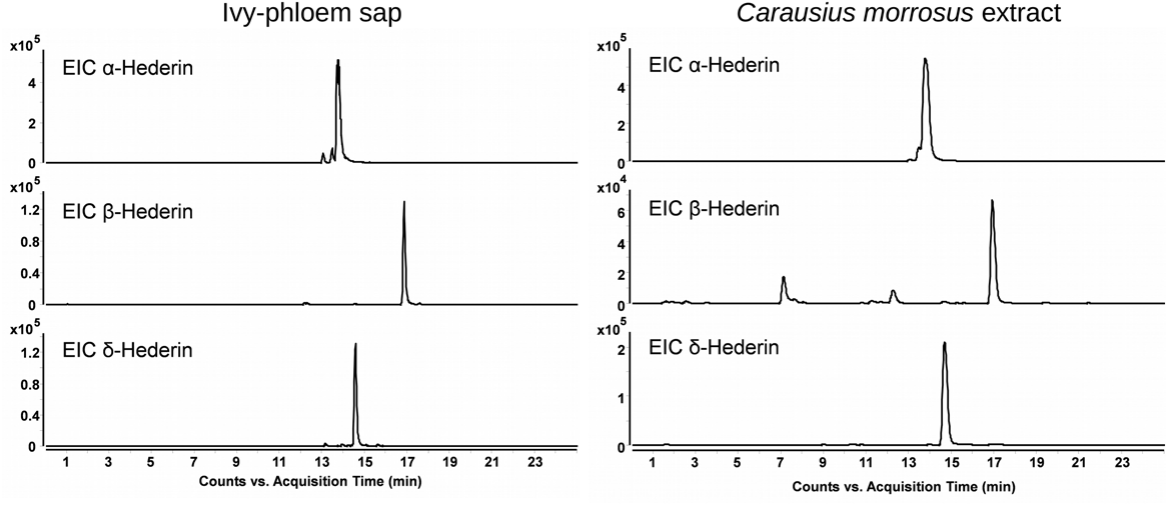
**Saponins can be found in** **ivy phloem sap as well as in** **stick insects fed on ivy.** Left: HPLC-MS analysis of 1 µl of ivy phloem sap filled up to 100 µl with insect ringer solution. Extracted ion chromatograms (EIC) of α-hederin ([M-H]^−^ *m/z* 749.4481+[M+HCOO]^−^ *m/z* 795.4536), β-hederin ([M-H]^−^ *m/z* 733.4532+[M+HCOO]^−^ *m/z* 779.4587) and δ-hederin ([M-H]^−^ *m/z* 603.3902+[M+HCOO]^−^ *m/z* 649.3957). Right: HPLC-MS analysis of a *C.* *morosus* extract. Extracted EIC of α-hederin ([M-H]^−^ *m/z* 749.4481+[M+HCOO]^−^ *m/z* 795.4536), β-hederin ([M-H]^−^ *m/z* 733.4532+[M+HCOO]^−^ *m/z* 779.4587) and δ-hederin ([M-H]^−^ *m/z* 603.3902+[M+HCOO]^−^ *m/z* 649.3957).

**Fig. 3.**
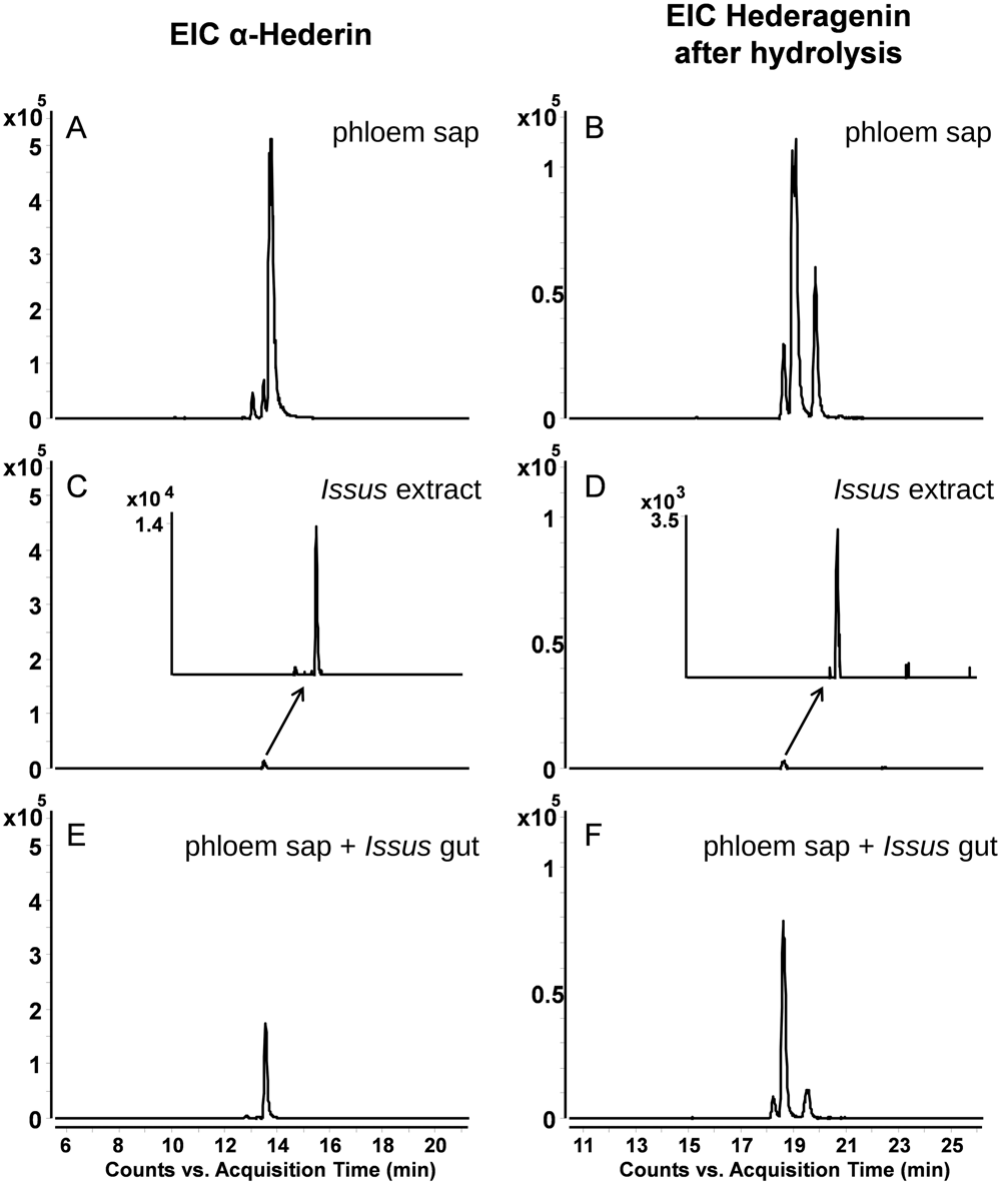
**α-hederin can be found in ivy phloem sap but not in** *I.* ***coleoptratus* and can be reduced significantly if** *I.* ***coleoptratus* gut and salivary glands are added to phloem sap.** EICs of α-hederin ([M-H]^−^ *m/z* 749.4481+[M+HCOO]^−^ *m/z* 795.4536) and hederagenine ([M-H]^−^ *m/z* 471.3480) are shown. (A) 1 µl of ivy phloem sap filled up to 100 µl with insect ringer solution. (B) 1 µl of ivy-phloem sap filled up to 100 µl with insect ringer solution after acidic hydrolysis. (C) *I.* *coleoptratus* extract. (D) *I.* *coleoptratus* extract after acidic hydrolysis. (E) Extracted salivary glands and the attached gut from one animal added to 1 µl of ivy phloem sap filled up to 100 µl with insect ringer solution. (F) Extracted salivary glands and the attached gut from one animal added to 1 µl of ivy phloem sap filled up to 100 µl with insect ringer solution after hydrolysis.

Thus we initially focused on ivy and the content of ivy-phloem sap which clearly yields intense signals for α-, β- and δ-hederin (Fig. 2) in HPLC-MS. Animals like the stick insect *Carausius morosus* fed with ivy become poisonous themselves and must not be used as feed for other arthropods or reptiles (own unpublished painful observations). When analysing *C.* *morosus* nymphs fed on ivy and of a weight comparable to an adult *I.* *coleoptratus*, clear and very large saponin peaks can be found (Fig. 2). This is in contrast to an extraction of *I.* *coleoptratus* (whole animal shock frozen and extracted) which, when compared to the phloem sap (Fig. 3A,B), hardly contains any detectable trace of saponins (Fig. 3C) and even the hederagenine is missing after acidic hydrolysis (Fig. 3D). Taken together, ivy contains saponins; when stick insects are fed on this plant they accumulate saponins in their bodies. By contrast when *I.* *coleoptratus* feeds on ivy it does not accumulate them.

So how does *I. coleoptratus* cope with the saponins? To find out if the large salivary glands and the long gut are causally involved, we collected phloem sap and added the excised salivary glands and the attached gut from one animal to 1 µl of ivy-phloem sap filled up to 100 µl with insect ringer solution. As control 1 µl of the same phloem sap sample was only filled up with the equal amount of ringer solution. The samples were kept for at least 1 h at room temperature, then stored over night at 4°C and then subjected to HPLC-MS. All experiments were repeated at least twice and the repetitions showed identical results. As can be seen in Fig. 3E,F α-hederin is reduced by over 80% and the amount of hederogenine after acidic hydrolysis is reduced significantly by over 50%. The results in Fig. 3 depict the values for α-hederin, however, all other saponins from ivy yield comparable results (β- and δ-hederin reduced by 74% and 83%, respectively), i.e. the saponins and genines in the animal are close or below the detection limit and a dramatic reduction of the saponin and genine content of a phloem sap sample can be observed, when salivary glands and gut from *I.* *coleoptratus* are added.

These results clearly show that some components from the gut and/or the salivary glands can metabolise saponins. This is obviously a rather slow process as after an incubation time of 1 h at room temperature the detectable saponins have been reduced by about 80% and the core structure was reduced by 50%. The exact time line of this degradation process remains to be determined in future studies. It is tempting to speculate that the exceptionally long midgut might be causally involved in this degradation process.

It is surprising that the stabile genine, a triterpene, is also degraded. It remains to be determined which enzymes and/or other components like symbiotic micro organisms from the *I.* *coleoptratus* digestive tract are involved in saponin handling and if other terpenes like the poisonous substances from other potential host plants of *I.* *coleoptratus* can also be degraded.

## MATERIALS AND METHODS

### Phloem sap

Ivy (*Hedera helix*) was harvested from ivy patches near the university of Linz (Austria, 48°20′N 14°19′E) in the morning, preferably after night rain. Fine glass-capillaries with tip diameters of approximately 50-200 µm were pulled by hand from Ø 0.9 mm glass tubes (Hartenstein, Würzburg, Germany) over a Bunsen burner. Ivy stems were cut and the exposed surface was gently touched with the capillary which caused phloem sap to enter it by capillary forces. The acquired phloem sap was blown out into a 0.5 ml Eppendorf vial.

### Animals

*Issus coleoptratus* adults were collected from June to August near the University of Linz and kept until use on ivy plants in a terrarium at room temperature.

For preparation, *I. coleoptratus* were kept at 4°C for about 20 min prior to the experiment. Then the animals were submerged in 4°C cold insect ringer solution containing 8.2 g/l NaCl, 0.75 g/l KCl, 0.4 g/l NaHCO_3_, 0.45 g/l Na_2_HPO_4_×H_2_O, 0.4 g/l MgCl×6H_2_O, 0.3 g/l CaCl_2_×2H_2_O and 5 g/l glucose (all purchased from Lactan, Graz, Austria) and fixed with needles. The insects were opened by a longitudinal cut along the dorsal midline. Gut and salivary glands were removed without delay in order to avoid washing out of components and transferred into Eppendorf vials containing 1 µl of phloem sap and 100 µl of ringer solution.

*C.* *morosus* served as control animals as they also feed on ivy. They were kept in a lab colony and fed exclusively with ivy. Young nymphs were taken for comparison which had approximately the same weight as adult *I.* *coleoptratus*.

For saponin extraction, the insects (*I.* *coleoptratus* and *C.* *morosus*) were frozen at −20°C for 1 h and crushed in a mortar. The crushed tissues from one animal were covered by 100 µl water-ethanol (50% v/v) and extracted at 4°C overnight.

### HPLC-MS

Chromatography was performed on an Agilent Series 1100 HPLC system equipped with vacuum degasser, quaternary pump, autosampler (10 µl injection volume), and UV-vis diode array detector (all from Agilent, Palo Alto, CA, USA). The separation column was a Phenomenex Kinetex C18–50×4.6 mm, 2.6 µm particle size. The mobile phase consisted of a gradient of water and acetonitrile with a constant concentration of 0.1% formic acid over the whole run. Initial conditions were 25% acetonitrile and the gradient was run to 90% acetonitrile within 28 min. The flow rate was set to 0.5 ml min^−1^.

Mass spectrometry detection was performed on an Agilent 6520 QTOF with electrospray ionisation (ESI) in the negative mode. The following ion source conditions were used: drying gas temperature 350°C, drying gas flow 11 l min^−1^, nebuliser pressure 55 psi, fragmentor voltage 200 V and capillary voltage 4000 V.

### Hydrolysis

The sample was acidified by adding concentrated hydrochloric acid (final concentration 3 M HCl) and heated at 90°C for 30 min in a closed vial (Elias et al., 1991). The solution was then neutralised with solid sodium carbonate, centrifuged at 16000 ***g*** and the supernatant was injected into the HPLC system.
